# Two-year results of disease activity score (DAS)-remission-steered treatment strategies aiming at drug-free remission in early arthritis patients (the IMPROVED-study)

**DOI:** 10.1186/s13075-015-0912-y

**Published:** 2016-01-21

**Authors:** Lotte Heimans, Gülşah Akdemir, Kirsten V. C. Wevers-de Boer, Yvonne P. Goekoop-Ruiterman, Esmeralda T. Molenaar, Johannes H. L. M. van Groenendael, Andreas J. Peeters, Gerda M. Steup-Beekman, Leroy R. Lard, Peter B. J. de Sonnaville, Bernard A. M. Grillet, Tom W. J. Huizinga, Cornelia F. Allaart

**Affiliations:** Department of Rheumatology, Leiden University Medical Center, P.O. BOX 9600, Leiden, 2300 RC The Netherlands; Department of Rheumatology, Haga Hospital, The Hague, The Netherlands; Department of Rheumatology, Groene Hart Hospital, Gouda, The Netherlands; Department of Rheumatology, Franciscus Hospital, Roosendaal, The Netherlands; Department of Rheumatology, Reinier de Graaf Gasthuis, Delft, The Netherlands; Department of Rheumatology, Bronovo Hospital, The Hague, The Netherlands; Department of Rheumatology, MCH Antoniushove, Leidschendam, The Netherlands; Department of Rheumatology, Admiraal de Ruyter Ziekenhuis, Goes, The Netherlands; Department of Rheumatology, Zorgsaam, Terneuzen, The Netherlands

**Keywords:** Rheumatoid arthritis, Methotrexate, Remission steered treatment, Radiology, Joint damage, Progression

## Abstract

**Background:**

Early suppression of disease activity in (rheumatoid) arthritis (RA) patients may result in drug-free remission and prevent damage. We assessed 2-year clinical and radiological outcomes of two disease activity score (DAS)-remission-steered treatment strategies in early arthritis patients.

**Methods:**

Patients (n = 610) with early RA or undifferentiated arthritis (UA) were treated with methotrexate (MTX) and tapered high dose of prednisone. Patients in early remission (44/53 joints DAS <1.6) after 4 months tapered and stopped medication. Patients who did not achieve early DAS-remission were randomized to either MTX plus hydroxychloroquine plus sulphasalazine plus low dose prednisone (arm 1) or to MTX + adalimumab (arm 2). At four-monthly intervals, medication was tapered and stopped if DAS was <1.6 but restarted, increased or switched if DAS was ≥1.6. Proportions of (drug-free) DAS-remission (DFR) after 2 years and Sharp-van der Heijde scores (SHS) were analyzed separately for the treatment strategies and patients with RA and UA.

**Results:**

After 2 years, 301/610 (49 %) patients were in DAS-remission and 131/610 (21 %) in DFR. In the early remission group 241/387 patients (62 %) were in DAS-remission and 111/387 (29 %) DFR. In arm 1 22/83 (27 %) and in arm 2 24/78 (31 %) were in DAS-remission, and 6/83 (7 %) and 7/78 (9 %), respectively, were in DFR. RA and UA patients achieved DAS-remission in comparable percentages (RA: 234/479 (49 %), UA: 64/122 (52 %), p = 0.25). More UA patients achieved DFR (41/122 (34 %)) compared to RA patients (89/479 (19 %), p<0.001). Mean (SD) DAS over time was 1.74 (0.58) across all patients, and median (IQR) SHS progression was 0 (0–0).

**Conclusions:**

After 2 years remission-steered treatment in early RA and UA patients, DAS-remission and DFR percentages were relatively low. Patients who achieved early remission more often achieved (drug-free) remission after 2 years than patients who needed additional treatment steps in the randomization arms, and more UA than RA patients achieved DFR. Overall, disease activity and radiologic damage progression in all patients were well suppressed.

**Trial registration:**

http://www.controlled-trials.com/ISRCTN11916566 Registered 07/11/2006 and EudraCT number 2006-06186-16 Registered 16/07/2007.

**Electronic supplementary material:**

The online version of this article (doi:10.1186/s13075-015-0912-y) contains supplementary material, which is available to authorized users.

## Background

In recent decades the treatment of rheumatoid arthritis (RA) has considerably changed, aiming at earlier suppression of disease activity, resulting in better outcomes [1–3]. The need to start disease-modifying antirheumatic drugs (DMARDs) earlier is incorporated through new classification criteria for RA [4], which include patients in earlier phases of the disease process. In addition, several trials have included or focused on patients with arthritis not (yet) fulfilling these criteria (undifferentiated arthritis (UA)) [5–13]. It has become clear that treatment to target prevents gradual deterioration [14, 15]. Based on results of clinical trials with treatment to target, in which a large percentage of RA patients achieved clinical remission [16–18], it is suggested that remission should be the treatment target [15]. Several trials [5, 16–20] have shown that initial treatment with a combination including methotrexate (MTX) and corticosteroids results in earlier suppression of inflammation and damage progression. It is hypothesized that induction of early remission may prevent chronicity of arthritis and allow tapering of treatment to drug-free remission (DFR) [21]. In UA this may be even more readily achieved, although in the PROMPT study, monotherapy with MTX proved insufficient to permanently induce remission in patients with UA [10].

The IMPROVED study was designed following the intention and results of these studies. It aims to achieve early clinical remission, followed by tapering of medication to DFR. Patients both with early RA (based on the new classification criteria) and with UA were included, and treated according to the same protocol, starting with a combination of MTX with prednisone, then tapering or adding DMARDs, depending on whether treatment-target clinical remission has been achieved. In this secondary analysis of the IMPROVED-study, the clinical and radiological outcomes of 2 years of remission-targeted treatment are presented.

## Methods

### Study design

The IMPROVED study (ISRCTN11916566 and EudraCT number 2006-06186-16) is a multicentre two-step randomized single-blinded clinical trial designed by Dutch rheumatologists participating in the Foundation for Applied Rheumatology Research (FARR). Patients were recruited between March 2007 and September 2010 in 12 hospitals in the Western part of the Netherlands. The study protocol was approved by the Medical Ethics Committee of each participating centre (listed in “Acknowledgements”).

### Patients

Patients were ≥18 years old, with early RA or UA, a disease activity score (DAS) ≥1.6, and no previous antirheumatic therapy. RA was defined as fulfilling the 2010 American College of Rheumatology (ACR) and European League Against Rheumatism (EULAR) classification criteria [4] with a symptom duration ≤2 years. UA was defined as at least one joint with clinical synovitis and one other painful joint, clinically suspected as due to early RA, regardless of symptom duration. Exclusion criteria were previously published [13, 22]. All patients gave written informed consent.

### Intervention

**Fig. 1:**
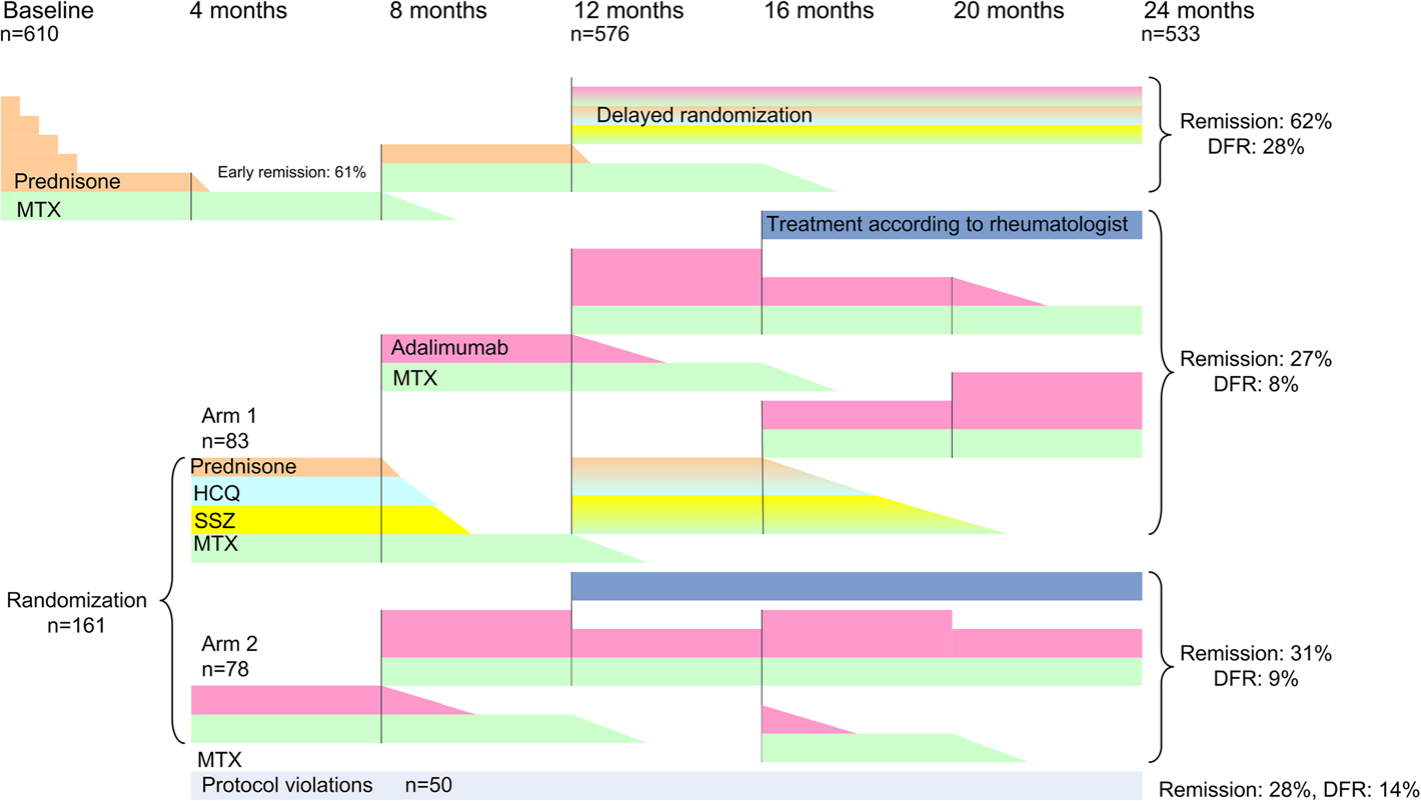
Study flow chart with percentages disease activity score (DAS) and drug-free remission after the second study year. *Orange* prednisone, *green* methotrexate (MTX), *dark blue* treatment according to opinion of rheumatologist (TAR), *aqua* hydroxychloroquine (HCQ), *yellow* sulfasalazine (SSZ), *purple* adalimumab biweekly, *double thickness purple* adalimumab weekly, *grey* protocol not followed as required but remained in follow up (outside of protocol, OOP). *DFR* drug-free remission

All patients started with 4 months of MTX 25 mg/week and prednisone 60 mg/day tapered to 7.5 mg/day in 7 weeks. Every 4 months the DAS (based on a 44-swollen-joint count and the Richie articular index, both including the feet) [23] was assessed by a trained research nurse, blinded for treatment allocation. The treatment target of the study was a DAS <1.6, which was considered to denote remission (Fig. 1) [24].

Patients in early remission (DAS <1.6 after 4 months) tapered prednisone with in 3 weeks with a dose reduction of 2.5 mg/day each week to 0 mg/day. When still in remission after 8 months, MTX was also tapered to 0 within 10 weeks (every week tapered with 2.5 mg/wk). In patients with a DAS ≥1.6 after 8 months, prednisone was restarted at 7.5 mg/day.

Patients with a DAS ≥1.6 after 4 months were randomized, either to hydroxychloroquine (HCQ) 400 mg/day and sulphasalazine (SSZ) 2000 mg/day added to MTX and prednisone (arm 1), or they switched to MTX 25 mg/week plus adalimumab (ADA) 40 mg/2 weeks (arm 2). Patients who had achieved early remission and discontinued prednisone, then lost remission and restarted prednisone without achieving remission, were also randomized to arm 1 or 2 (delayed randomization) (Fig. 1). In arm 1, if remission after 8 months was achieved, prednisone, SSZ and then HCQ were stopped. MTX was stopped if remission remained 4 months later. If remission was not achieved at 8 months, patients switched to MTX + ADA (40 mg/2 weeks, increased to 40 mg/week if DAS remained ≥1.6). Patients in arm 2 tapered ADA in they were in remission after 8 months, and increased ADA to 40 mg/week if there was no remission. The weekly dose of ADA (in combination with MTX) was exploratory and is not evidence based. Based on the costs of medication, and in view of a subsequent report on dose-dependent risk of side effects, ADA 40 mg/week is not approved in current daily practice [25].

In both arms, if patients did not achieve remission on a combination of MTX + ADA 40 mg/week, further treatment decisions were left to the opinion of the rheumatologist (Fig. 1). A detailed description of the randomization procedure was previously published [22].

### Primary and secondary outcomes

Primary outcomes were percentages of patients in DAS-remission and DFR based on a DAS <1.6. Secondary outcomes were DFR based on the proposed remission definition published by the ACR/EULAR in 2011 (Boolean) [26] mean DAS, mean functional ability as measured by the Dutch version of the health assessment questionnaire (HAQ) [27], radiological evidence of damage progression in the joints of the hands and feet (defined as an increase ≥0.5 points in the Sharp-van der Heijde score (SHS)) [28] and toxicity.

Baseline and yearly radiographs of the hands and feet were anonymized and scored in time-random order for the presence of erosions and joint space narrowing, by two trained, independent readers (LH and GA). Only 8 % of the patients had progression and therefore intra-class correlation coefficients were not suitable for measuring reliability [29]. In 443 of 496 patients who had radiographs taken after 2 years follow up, there was an inter-reader difference <2 between the progression scores of both readers. Consensus on score was reached for the other 53 patients.

Outcomes were reported separately for patients who achieved early DAS-remission and those randomized and were compared between the randomization arms, and between RA and UA patients, and between patients who were in remission or not in remission after 2 years.

Treatment during 2 years was plotted in a figure as percentages of patients on medication per treatment group. The figure shows not treatment steps, but actual medication use, with medications categorized as ‘other’ being those that were prescribed either according to the protocol after failure on ADA (treatment according to physician) or outside the regular treatment steps, although still DAS-remission-steered (outside of protocol).

### Statistical analysis

We performed intention-to-treat analyses. Outcomes were compared using Student’s *t* test, the Mann–Whitney *U* test and the chi square (*χ*^2^) test. DAS and HAQ over time were compared using linear mixed models (LMM), with treatment strategy (arms 1 and 2) and time (study visit) as fixed effects in an unstructured covariance structure. All statistical analyses were conducted with SPSS for Windows version 20.0.

## Results

### Study population

Of the 610 patients, 479 (79 %) had classifiable RA (2010 criteria) and 122 (20 %) UA (9 patients could not be classified because of missing data). Of 610 patients, 387 (63 %) achieved early DAS-remission at 4 months (early remission group). Of the 610 patients, 161 (26 %) with DAS ≥1.6 at 4 months were randomized, 83 patients to arm 1 and 78 to arm 2. Fifty patients with a DAS ≥1.6 at 4 months were not randomized because the treating physician declared the patient in clinical remission. These patients were analyzed in the outside-of-protocol (OOP) group. Twelve patients left the study before the assessment at 4 months (Table 1). Over 2 years 79 patients were lost to follow up; 54 withdrew consent, 9 discontinued because of a revised diagnosis and 8 because of co-morbidity. Eight patients died [13, 22], of whom three died in the second year of the study (Additional file 1).

**Table 1:** Baseline characteristics of the IMPROVED study population

	Total n = 610	Early remission n = 387	Arm 1 n = 83	Arm 2 n = 78	OOP n = 50
DAS, mean ± SD	3.2 ± 0.9	3.0 ± 0.8	3.6 ± 0.9	3.6 ± 1.0	3.6 ± 0.9
HAQ, mean ± SD	1.2 ± 0.7	1.0 ± 0.7	1.4 ± 0.6	1.4 ± 0.6	1.3 ± 0.7
Age in years, mean ± SD	52 ± 14	52 ± 14	49 ± 14	51 ± 14	54 ± 14
Female, n (%)	414 (68)	240 (62)	64 (77)	58 (74)	42 (84)
Symptom duration (weeks), median (IQR)	18 (9–32)	17 (9–30)	22 (9–41)	21 (8–31)	18 (9–42)
RF-positive, n (%)	339 (56)	224 (58)	41 (49)	43 (55)	23 (46)
ACPA-positive, n (%)	333 (55)	225 (58)	40 (48)	37 (47)	25 (50)
RA (2010 criteria), n (%)	479 (79)	298 (77)	66 (80)	66 (85)	40 (80)
Swollen joint count, median (IQR)	5 (3–10)	5 (2–9)	6 (3–10)	8 (4–12)	7 (3–13)
Tender joint count, median (IQR)	6 (4–9)	5 (3–8)	8 (6–13)	9 (6–13)	8 (6–14)
ESR mm/h, median (IQR)	25 (11–39)	23 (8–38)	28 (13–41)	22 (11–41)	29 (16–42)
VAS global health (mm), mean ± SD	46 ± 23	43 ± 24	53 ± 20	54 ± 22	49 ± 23
Total SHS, median (IQR)	0 (0–1.0)	0 (0–0.5)	0 (0–0)	0 (0–0)	0 (0–0)
Erosive, n (%)	89 (15)	63 (16)	10 (12)	13 (17)	3 (6)

After 4 months 12 patients were lost to follow up and 598 patients were categorized as described in Table 1. Arm 1: randomized at 4 months to methotrexate, sulphasalazine, hydroxychloroquine and low-dose prednisone. Arm 2: randomized at 4 months to methotrexate and adalimumab. *OOP* outside of protocol, *SD* standard deviation, *IQR* interquartile range, *n* number, *DAS* disease activity score, *HAQ* health assessment questionnaire, *RF* rheumatoid factor, *ACPA* anti-citrullinated protein antibodies, *RA (2010)* rheumatoid arthritis according to the 2010 classification criteria, *ESR* erythrocyte sedimentation rate, *VAS* visual analogue scale, *SHS* Sharp-van de Heijde score, *Erosive* at least 1 erosion

### DAS-remission and drug-free remission

Of the 610 patients, 55 (9 %) (37 with RA, 17 with UA, *p*=0.01, and 1 patient who was unclassifiable because of missing data) were in sustained DAS-remission from 4 months through to 2 years and therefore, in DFR from 8 months to 2 years. There were 50 patients (8 %) who never achieved DAS-remission during 2 years of follow up. Medication was reintroduced in patients who achieved DAS-remission but lost it again after drug tapering. At the next evaluation, 75 % of those patients were again in DAS-remission. At the time point (t) = 2 years, 301/610 (49 %) patients were in DAS-remission and 131/610 (21 %) were in DFR. In the early remission group, 241/387 (62 %) were in DAS-remission and 111/387 (29 %) in DFR at t = 2 years. There were 22/83 patients (27 %) in arm 1 and 24/78 (31 %) in arm 2 in DAS-remission (*p*=0.76), and 6/83 patients (7 %) in arm 1 and 7/78 patients (9 %) in arm 2 were in DFR at t = 2 years (*p*=0.73). Finally, at t = 2 years, 138 of all 610 patients (23 %) were in ACR/EULAR remission (Boolean), with 117/387 (30 %) in the early remission group, 2/83 (2 %) in arm 1, and 14/78 (18 %) in arm 2 (arm 1 versus arm 2, *p*=0.001).

At t = 2 years, comparable percentages of anti-citrullinated protein antibody (ACPA)-positive and ACPA-negative patients were in DAS-remission (ACPA-positive 172/333 (52 %), ACPA-negative 125/262 (48 %), *p*=0.68) but more ACPA-negative patients achieved DFR than ACPA-positive patients: 74/262 (28 %) versus 54/333 (16 %), *p*<0.001. Comparable percentages of UA or RA patients achieved remission after 2 years (UA 64/122 (52 %) and RA 234/479 (49 %), *p*=0.25), but significantly more UA patients, of whom 94 % were ACPA-negative, achieved DFR (41/122 (34 %) compared to 89/479 (19 %) in RA patients, *p*<0.001) (Additional file 1: Table S1).

### DAS and HAQ after 2 years

Patients in DAS-remission at 2 years had a mean (SD) HAQ of 0.29 (0.39) compared to 0.94 (0.63) in patients who were not in remission (*p*<0.001), and a mean (SD) DAS of 0.92 (0.38), compared to 2.32 (0.57) in patients who were not in DAS-remission (*p*<0.001). This resulted from significant differences in both subjective and (semi-)objective DAS components. Symptom duration at inclusion was not related to achieving or not achieving DAS-remission at t = 2 years. Of 204 patients who at baseline had <12 weeks symptom duration, 106 (52 %) were in DAS-remission and 50 (25 %) were in DFR at 2 years, compared to 192/397 (50 %) (*p*=0.31) and 80/397 (20 %) (*p*=0.19) of those who had had symptoms for ≥12 weeks.

**Fig. 2:**
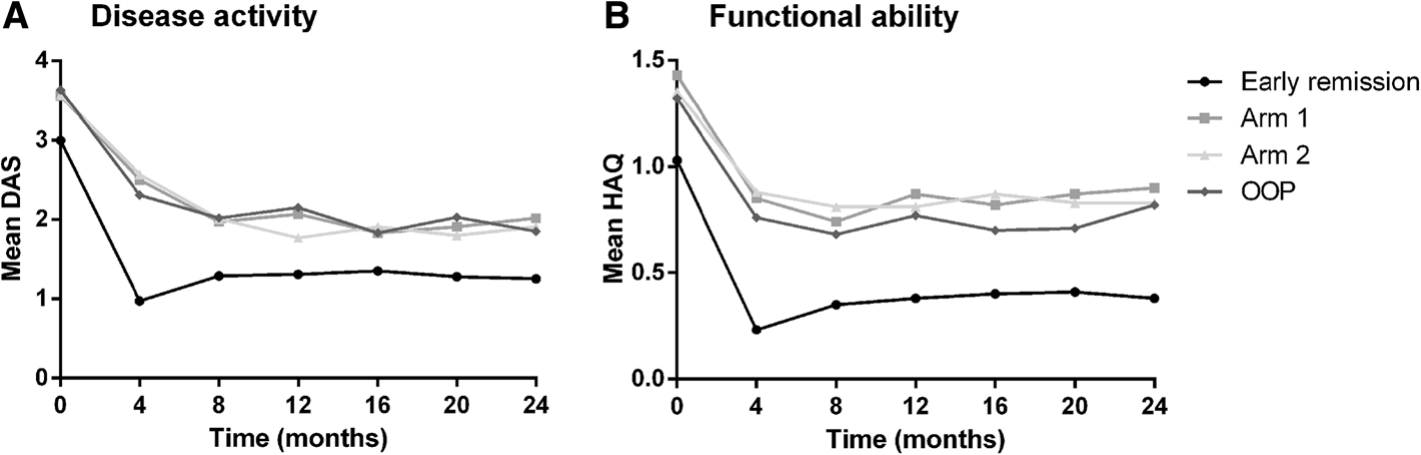
Mean disease activity score (*DAS*) and health assessment questionnaire (*HAQ*) according to treatment group during 2 years of follow up. *OOP* outside of protocol

For all patients mean (SD) DAS over time was 1.74 (0.58) and mean HAQ 0.61 (0.47). In the early remission group this was 1.25 (0.77) and 0.38 (0.48), in arm 1 it was 2.02 (0.70) and 0.9 (0.66), and in arm 2 it was 1.92 (0.85) and 0.83 (0.67) (Table 2 and Fig. 2). Over time, neither DAS nor HAQ were significantly different between arms 1 and 2 (mean difference (95 % CI) LMM for DAS 0.01 (−0.2, 0.2) and for HAQ 0.1 (−0.1, 0.2)) (Fig. 2).

**Table 2:** Outcomes in the IMPROVED-study population after 2 years

	Total n = 610	Early remission n = 387	Arm 1 n = 83	Arm 2 n = 78	*P* value arm 1 vs 2	OOP n = 50
DAS, mean ± SD	1.5 ± 0.8	1.3 ± 0.8	2.0 ± 0.7	1.9 ± 0.9	0.45	1.9 ± 0.7
HAQ, mean ± SD	0.5 ± 0.6	0.4 ± 0.5	0.9 ± 0.7	0.8 ± 0.7	0.55	0.8 ± 0.7
Swollen joint count, median (IQR)	0 (0–1)	0 (0–1)	1 (0–2)	0 (0–2)	0.25	0 (0–2)
Tender joint count, median (IQR)	1 (0–3)	0 (0–2)	3 (2–5)	3 (1–6)	0.84	2 (1–4)
ESR mm/h, median (IQR)	9 (5–17)	8 (4–16)	11 (6–20)	9 (6–17)	0.19	14 (7–25)
VAS global health (mm), mean ± SD	22 ± 22	18 ± 21	30 ± 21	28 ± 24	0.61	32 ± 22
Total SHS, median (IQR)	0 (0–0.5)	0 (0–0.5)	0 (0–1.1)	0 (0–0)	0.12	0 (0–0.3)
Erosive, n (%)	50 (8)	39 (10)	2 (2)	8 (10)	0.04	1 (2)
SHS progression, n (%)	50 (8)	33 (9)	9 (11)	5 (6)	0.31	3 (6)
DAS-remission, n (%)	301 (49)	241 (62)	22 (27)	24 (31)	0.76	14 (28)
Drug-free remission, n (%)	131 (22)	111 (29)	6 (7)	7 (9)	0.73	7 (14)
ACR/EULAR remission, n (%)	138 (23)	117 (30)	2 (2)	14 (18)	0.001	5 (10)

After 4 months 12 patients were lost to follow up and 598 patients were categorized. Arm 1: randomized after 4 months to methotrexate, sulphasalazine, hydroxychloroquine and low-dose prednisone. Arm 2: randomized after 4 months to methotrexate and adalimumab. *OOP* outside of protocol, *SD* standard deviation, *IQR* interquartile range, *n* number, *DAS* disease activity score, *HAQ* health assessment questionnaire, *ESR* erythrocyte sedimentation rate, *VAS* visual analogue scale, *SHS* Sharp-van de Heijde score, *Erosive* at least 1 erosion, *Progression* increase in SHS ≥0.5 points, *DAS-remission* DAS <1.6 [24], *ACR/EULAR remission* provisional Boolean-based remission definition published by the American College of Rheumatology and the European League Against Rheumatism based on a 44-joint count [26]

### Radiological joint damage

Median SHS progression in all groups was 0 (range 0–22). Only 50/610 (8 %) patients had radiological progression defined as an increase in SHS ≥0.5; in the early remission group there were 33/387 (9 %) patients with progression, in arm 1 there were 9/83 (11 %), in arm 2 there were 5/78 (6 %) (arm 1 versus arm 2, *p*=0.31), and in the OOP group there were 3/50 (6 %). There was no significant difference in progression score between patients who were in DAS-remission at 2 years and patients who were not. Of the 610 patients, 8 (1 %) had radiological evidence of damage progression in ≥5 points after 2 years, which represents the minimal clinically important difference [30]. Seven of these eight patients were in early remission after 4 months and tapered prednisone to zero, after which five patients relapsed, needing to restart prednisone. One patient did not achieve early remission and was randomized to arm 2. After 2 years, erosions were seen on radiographs of the hands or feet in 39/387 (10 %) of patients in the early remission group, in 2/83 (2 %) in arm 1, in 8/78 (10 %) in arm 2 (arm 1 versus arm 2, *p*=0.04), and in 1/50 (2 %) of patients in the OOP group.

### Therapy

**Fig. 3:**
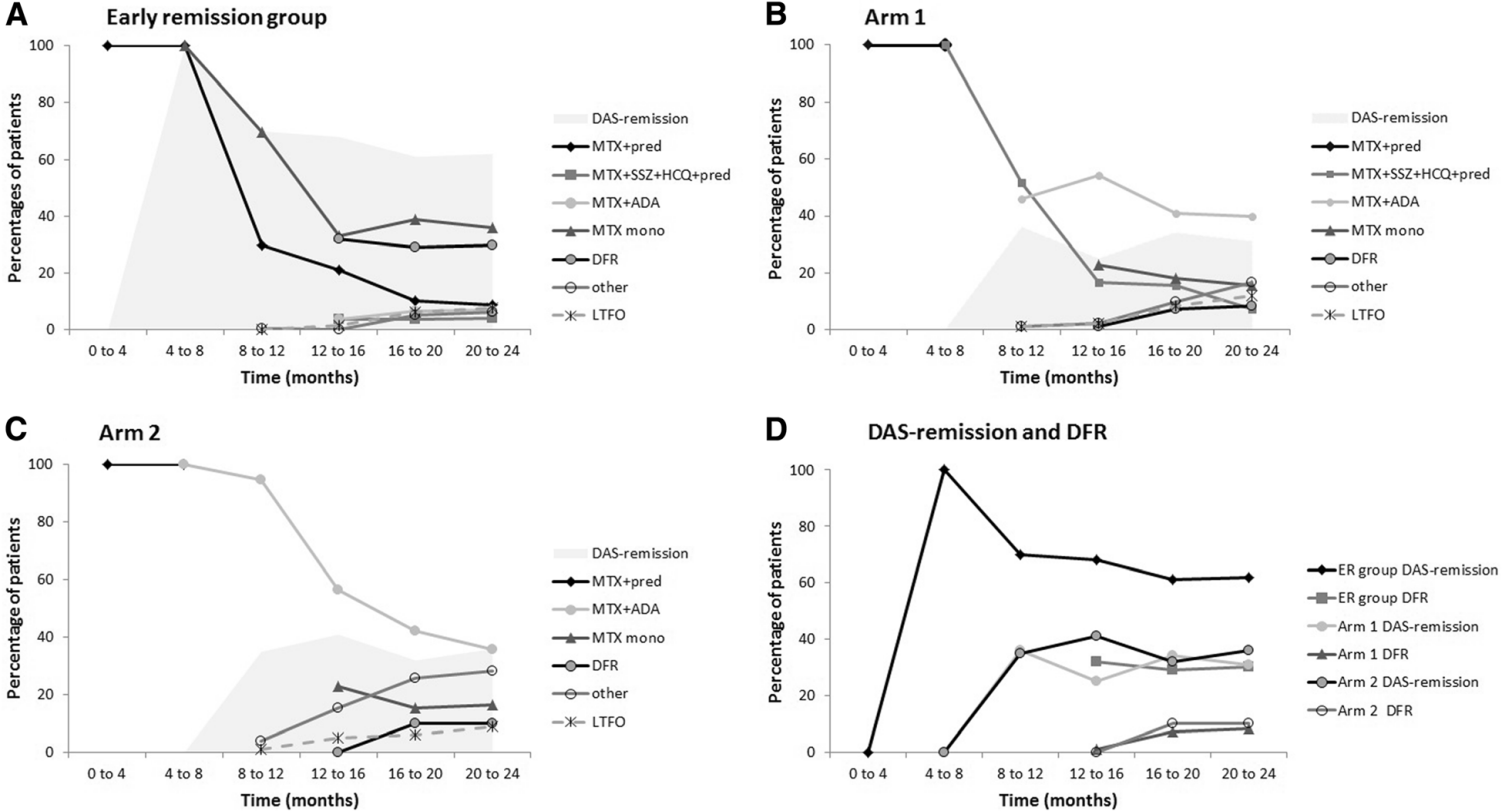
Treatment over time in the early remission group (**a**), Arm 1 (**b**) and Arm 2 (**c**), in percentage of total per treatment group; percentages in disease activity score (DAS)-remission and percentages in drug-free remission (DFR) (**d**). **a** Early remission group; **b** Arm 1; **c**. Arm 2. *Lines* are approximations of the proportions of patients discontinuing medication (according to tapering strategies or due to side effects), or starting medications according to DAS-remission-steered escalation strategies, across various treatment steps per arm, over time. The category *Other* includes medications that were prescribed per protocol in the ‘treatment according to rheumatologist’ step after failure on methotrexate plus adalimumab, and medications prescribed outside of the protocol but still maintaining a DAS-remission-targeted strategy. *Shaded areas* denote patient proportions in DAS-remission over time. **d**. Proportions of patients in DAS-remission and DFR per strategy over time. *MTX* methotrexate, *pred* prednisone, *SSZ* sulphasalazine, *HCQ* hydroxychloroquine, *ADA* adalimumab, *mono* monotherapy, *DFR* drug-free (DAS) remission, *LTFO* lost to follow up, *ER* early remission

The percentages of patients on various medications according to the prescribed treatment steps per 4 months in the early remission group, in arm 1, and in arm 2 are depicted in Fig. 3. In the early remission group treatment with prednisone decreased from 100 % of patients at treatment start to less than 10 % at t = 2 years (Fig. 3a). Having all also started with MTX treatment, 45 % still used MTX at t = 2 years. In the early remission group 15 % of patients, after having lost DAS-remission, did not regain DAS-remission after restart of prednisone, and were randomized to arm 1 or 2.

In arm 1, over 2 years of treatment 52/83 (63 %) patients failed to achieve DAS-remission on the combination of MTX with sulfasalazine, hydroxychloroquine and prednisone and started on adalimumab (with MTX), and up to 39 % of these increased adalimumab to once weekly by protocol. Over time, most patients discontinued adalimumab, but due to late switchers and also restarters because of DAS ≥1.6, after 2 years 40 % of patients in arm 1 were using adalimumab. In arm 2, 40 % of patients randomized to treatment with adalimumab initially increased the dose to 40 mg/week at month 8. The percentage of patients on adalimumab decreased during 2 years of treatment to 36 % (Fig. 3c), despite this. The main difference between arms 1 and 2 thus constitutes the higher initial use of adalimumab in arm 2, while adalimumab use levelled out to around 40 % of patients at year 2 in both arms. In addition, more patients in arm 2 progressed to other medications. No details are available for the OOP group, in whom treatment remained steered at remission, but with medication not as prescribed in the protocol.

### Toxicity

Details on toxicity in year 1 were reported previously [22], showing no significant differences between the treatment arms. During the second year of the study, 337/610 (55 %) patients reported 704 adverse events (AE), 53 % of the patients in the early remission group, 64 % in arm 1, 67 % in arm 2 (arm 1 versus arm 2, *p*=0.71), and 54 % in the OOP group. The most common AE were gastrointestinal complaints, upper airway infections, and skin rashes (Table 3). Twenty-five serious adverse events (SAE) were reported in the early remission group, five in arm 1, eight in arm 2, and three in the OOP group (Additional file 2: Table S2).

**Table 3:** Number of adverse events reported between 1 year and 2 years

	Early remission n = 387	Arm 1 n = 83	Arm 2 n = 78	OOP n = 50
Patients with AE*, n (%)	205/387 (53 %)	53/83 (64 %)	52/78 (67 %)	27/50 (54 %)
Total number of AE	408	129	109	58
Cardiovascular	25	5	8	4
Pulmonary	17	5	2	2
Gastrointestinal	67	16	14	12
GI complaints	8	2	2	–
Nausea/emesis	23	2	4	4
Increased liver enzymes	15	7	3	4
Other	21	5	5	4
Neuro-psychiatric	37	5	7	3
Headache	14	–	4	1
Dizziness	7	1	–	1
Mood disorders	4	–	–	1
Other	12	4	3	–
Urogenital	7	3	2	3
Skin/mucous membranes	45	18	15	3
Rash	19	8	5	–
Hair loss/thinning	4	1	1	1
Sicca complaints	3	–	–	1
Eczema	3	1	–	–
Other	16	8	9	1
Infections	106	38	41	18
Upper airway tract	29	11	16	10
Gastrointestinal	4	1	–	2
Skin/mucosa	14	2	4	2
Pneumonia/bronchitis	9	1	1	1
Urinary tract	15	7	5	1
Influenza/unspecified fever	25	10	6	1
Other	10	6	9	1
Trauma/injury	13	5	2	3
Infusion reaction	3	1	–	–
Malaise	9	5	1	1
Surgical procedures without hospitalization	13	5	4	1
Other	65	23	12	8

^*^One or more adverse events possible per patient. *OOP* outside of protocol, *AE* adverse events, *GI* gastrointestinal

## Discussion

Two years after initial therapy with MTX and a tapered high dose of prednisone, followed by DAS-remission-steered treatment including drug tapering and discontinuation, 49 % of the patients with early RA or UA were in DAS-remission, and 21 % were in DFR. Patients who achieved early DAS-remission after 4 months more often achieved DAS-remission over time (62 % at 2 years) and DFR (29 % at 2 years) than patients who did not (29 % DAS-remission and 9 % DFR at 2 years). There were no differences between the two treatment strategies. Mean DAS over time was significantly lower in the early remission group than in the other groups, but due to DAS-remission-steered treatment, mean (SD) DAS over time was low (1.74 (0.58)) across all patients. Radiological evidence of damage progression ≥0.5 SHS was seen in only 8 % of the patients and functional ability improved up to the normal range in those who achieved remission and was slightly lower in the other groups.

The study shows the effectiveness of early DAS-remission-steered therapy, resulting in low disease activity, improved functional ability and prevention of damage progression. In particular, the radiologic results are better than in previous studies such as the remission-steered NEO-RACo study in which 20–47 % of the patients had progression after 2 years, and better than in the DAS ≤2.4-steered BeSt study, in which 7–33 % of the patients across the four treatment arms had progression after 1 year [14, 17]. This is all the more remarkable as unlike in these studies, we introduced rapid tapering and discontinuation of medication in this study to aim for early DFR. The virtual absence of radiographic evidence of joint damage progression, which is mainly a pathophysiological important outcome as minimal damage has little clinical relevance [31], may be related to several trial aspects. We included patients with earlier disease and milder disease activity and less damage at baseline, and with fewer ACPA-positive patients than in the FIN-RACo, NEO-RACo and BeSt studies. As in the BeSt and COBRA study, we started treatment with a combination of high-dose MTX and a tapered high dose of prednisone, leaving out sulphasalazine, as in the COBRA-light study [20], which has subsequently shown that a lower dose of prednisone is not less effective than a high dose of prednisone. In addition, we designed the study to be able to introduce a TNF-inhibitor early in the disease course if DAS-remission was not achieved, also for patients with arthritis who were suspected to have early RA, who did not fulfil the classification criteria.

The clinical data are maybe not as spectacular. We had hypothesized that early remission-steered treatment including the initial high dose MTX and prednisone and option to expand or switch to multiple conventional synthetic (cs)DMARD or adalimumab, would result in induction of permanent remission in a large number of patients with early arthritis in this study population. Yet, the overall DAS-remission rates of 49 % in the IMPROVED study are lower than in the NEO-RACo study (60.5 %) [17] and only slightly higher than what we observed in the BeSt study (42 %) [14, 16].

The initial findings were promising. More than 60 % of patients achieved DAS-remission after 4 months of treatment, and of those, more than 30 % were in drug-free remission by the end of year 1, and 29 % were in drug-free remission after 2 years. Those not in early remission were randomized to two effective treatment options, with the hypothesis that earlier introduction of anti-TNF might result in more remission and better functional ability. After 1 year we found that patients in arm 2, who were randomized to treatment with adalimumab, achieved more DAS-remission than patients who were randomized to first try therapy with triple-csDMARDs plus low-dose-prednisone [22]. The weekly dose of adalimumab (in combination with MTX) was exploratory and is not evidence based. In current daily practice this is not approved, based on the costs of this medication and the dose-dependent risk of infection and malignancy [25]. Fortunately our patients who were treated with this combination of therapy did not have significantly more serious infections or malignancies.

After 2 years of DAS-remission-steered therapy, this difference was no longer evident. Similar results were found in the SWEFOT trial [32]. and the RACAT trial [33]. Initial improvement may depend on choice of initial therapy, but late outcomes depend on subsequent targeted treatment. Although delaying adalimumab in arm 1 may have delayed achieving DAS-remission in a proportion of patients, and there were only 2 patients in ACR/EULAR Boolean remission (compared to 14 in arm 2), this has had no relevant impact on radiologic outcomes, nor on the possibility to taper and stop adalimumab (Fig. 3). This is contrary to what we previously found in the BeSt study [16, 34, 35], in which delayed treatment with a TNF-inhibitor was associated with more maintained treatment with that TNF-inhibitor over time.

We were able to taper and stop medication in many patients, effectively avoiding prolonged use of prednisone and (although less so) adalimumab (Fig. 3) and achieving one in five patients being in drug-free (DAS) remission at year 2. However, more patients achieved remission and tapered medication; the majority then had a DAS >1.6 and medication had to be escalated again. It can be argued that we should not have tapered, or tapered and stopped the medication too fast. A longer induction treatment might have suppressed the disease more permanently. Drug-free remission in the BeSt study was introduced after up to 2 years of low disease-activity and 6 months of DAS-remission. Still 50 % of patients who achieved drug-free remission had to restart medication because of DAS ≥1.6 [36]. Continuous treatment during DAS-remission would have little impact on the radiological outcomes and could induce more side effects. In daily practice, one could consider tapering more slowly or having a maintenance dose, or substituting methotrexate with hydroxychloroquine before stopping completely, but we cannot support that with evidence.

We found that the overall remission rates were relatively low because patients who did not achieve early remission were also less likely to achieve remission later on. It might be that for the window of opportunity was already missed, even though the outcomes between patients with <12 weeks symptom duration and those with ≥12 weeks symptom duration were comparable. Remission rates and other outcomes (except for DFR) were also comparable between patients with RA and UA. Patients who did not achieve early remission may represent a selected group with more advanced and less responsive disease, who already had a higher HAQ score at baseline. Some of these patients may have had non-inflammatory symptoms or non-RA-related erythrocyte sedimentation rate (ESR) that may have influenced the DAS but would not respond to antirheumatic treatment. For these patients a DAS <1.6 may be unrealistic and remission-steered treatment adjustments may constitute overtreatment. By including and treating patients with UA we risked treating patients with a self-limiting non-RA type of arthritis, who would be among the patients who achieved DFR. At 2 years, 29 % of patients in the early remission group and 7–9 % in arms 1 and 2 were in DFR, which is a percentage not too different form the 25 % of UA patients who achieved spontaneous remission in the PROMPT trial [10]. We found that UA patients more often achieved DFR than RA patients, and anti-citrullinated protein antibody (ACPA)-negative patients more often than ACPA-positive patients. Interestingly, ACPA positivity was associated with achieving early DAS-remission at 4 months [13], and after 1 year of treatment, DAS-remission while on medication was achieved in RA and UA patients, and in ACPA-positive and ACPA-negative patients in comparable percentages. It appears that RA patients and ACPA-positive patients who achieved DAS-remission will more often flare when medication is tapered and stopped. This may affect future trial design and daily practice.

## Conclusion

After 2 years of remission-steered treatment in patients with early RA and UA, DAS-remission and DFR percentages were relatively low. Patients who achieved early remission more often achieved (drug-free) remission after 2 years than patients who needed additional treatment steps in the randomization arms, and more UA than RA patients achieved DFR. Damage progression as seen on radiographs was well suppressed in all patients. As suppression of radiologic damage progression is not enough, additional therapies, medicinal or other, should be investigated to improve clinical outcomes without risk of significant side effects, and further investigations should focus on identifying predictive factors or early markers of effective suppression of disease activity on the initial therapy, to choose the next treatment step and avoid delays in clinical response.

## Additional files

Additional file 1: Table S1. File shows baseline characteristics and clinical outcomes after 2 years of RA and UA patients. (DOC 49 kb) Additional file 2: Table S2. File shows serious adverse events during the second year of the IMPROVED study. (DOC 35.5 kb)

## Abbreviations

ACPA: anti-citrullinated protein antibodies
ACR: American College of Rheumatology
ADA: adalimumab
AE: adverse events
DAS: disease activity score
DFR: drug-free remission
DMARD: disease-modifying antirheumatic drug
ESR: erythrocyte sedimentation rate
EULAR: European League Against Rheumatism
FARR: Foundation for Applied Rheumatology Research
HAQ: health assessment questionnaire
HCQ: hydroxychloroquine
LMM: linear mixed models
MTX: methotrexate
OOP: outside of protocol
RA: rheumatoid arthritis
RF: IgM rheumatoid factor
SAE: serious adverse events
SHS: Sharp-van der Heijde score
SJC: swollen joint count
SSZ: sulphasalazine
TJC: tender joint count
TNF: tumour necrosis factor
UA: undifferentiated arthritis
VAS: visual analogue scale
