# Supplementary material for: Two-year results of disease activity score (DAS)-remission-steered treatment strategies aiming at drug-free remission in early arthritis patients (the IMPROVED-study)
Source: Arthritis Res Ther. 2016 Jan 21;18:23. doi: 10.1186/s13075-015-0912-y (PMC4721018; doi:10.1186/s13075-015-0912-y)
Supplement: Additional file 1: Table S1. — File shows baseline characteristics and clinical outcomes after 2 years of RA and UA patients. (DOC 49 kb) [file 13075_2015_912_MOESM1_ESM.doc]

Table S1. Baseline characteristics and clinical outcomes after 2 years of RA and UA patients.

|  | **RA** | **UA** |  |
| --- | --- | --- | --- |
|  | **n=479** | **n = 122** | **p-value** |
| **Baseline** |  |  |  |
| DAS, mean + SD | 3.3 ± 0.9 | 2.7 ± 0.7 | <0.001 |
| HAQ, mean + SD | 1.2 ± 0.7 | 1.0 ± 0.6 | 0.02 |
| Age in years, mean + SD | 52 ± 13 | 52 ± 16 | 0.90 |
| Female, n (%) | 333 (70) | 74 (61) | 0.06 |
| Symptom duration (weeks) , median (IQR) | 18 (9-34) | 16 (8-28) | 0.14 |
| RF positive, n (%) | 330 (69) | 5 (4) | <0.001 |
| ACPA positive, n (%) | 324 (68) | 4 (3) | <0.001 |
| Swollen Joint Count, median (IQR) | 7 (3-11) | 3 (2-6) | <0.001 |
| Tender Joint Count, median (IQR) | 7 (4-10) | 5 (3-8) | <0.001 |
| ESR mm/hr, median (IQR) | 26 (12-41) | 16 (9-38) | 0.01 |
| VAS global health (mm) , mean + SD | 48 ± 24 | 40 ± 21 | 0.001 |
| Total SHS, median (IQR) | 0 (0-0.5) | 0 (0-0.4 | 0.98 |
| Erosive, n (%) | 60 (13) | 12 (9) | 0.46 |
| **2 years** |  |  |  |
| DAS, mean + SD | 1.5 ± 0.8 | 1.3 ± 0.8 | 0.05 |
| HAQ, mean + SD | 0.5 ± 0.6 | 0.5 ± 0.6 | 0.88 |
| Swollen Joint Count, median (IQR) | 0 (0-1) | 0 (0-1) | 0.23 |
| Tender Joint Count, median (IQR) | 1 (0-3) | 1 (0-3) | 0.60 |
| ESR mm/hr, median (IQR) | 9 (5-19) | 7 (3-13) | 0.01 |
| VAS global health (mm) , mean + SD | 23 ± 23 | 20 ± 20 | 0.27 |
| Total SHS, median (IQR) | 0 (0-0.5) | 0 (0-0.5) | 0.88 |
| Erosive, n (%) | 42 (9) | 7 (6) | 0.34 |
| SHS progression, n (%) | 41 (9) | 9 (7) | 0.78 |
| DAS-remission, n (%) | 234 (49) | 64 (52) | 0.25 |
| Drug free remission, n (%) | 89 (19) | 41 (34) | <0.001 |
| ACR/EULAR remission, n (%) | 108 (23) | 29 (24) | 0.56 |
