# Supplementary material for: Two-year results of disease activity score (DAS)-remission-steered treatment strategies aiming at drug-free remission in early arthritis patients (the IMPROVED-study)
Source: Arthritis Res Ther. 2016 Jan 21;18:23. doi: 10.1186/s13075-015-0912-y (PMC4721018; doi:10.1186/s13075-015-0912-y)
Supplement: Additional file 2: Table S2. — File shows serious adverse events during the second year of the IMPROVED study. (DOC 35.5 kb) [file 13075_2015_912_MOESM2_ESM.doc]

|  | **Early remission**  **n=387** | **Arm 1**  **n=83** | **Arm 2**  **n=78** | **OOP**  **n=50** |
| --- | --- | --- | --- | --- |
| Patients with SAE, no (%) | 18/387 (5%) | 4/83 (5%) | 7/78 (9%) | 3/50 (6%) |
| Total number of SAE | 25 | 5 | 8 | 3 |
| **Died** | Cardiac arrest after pericarditis  Kidney failure during sepsis as complications of multiple myeloma | - | Pneumococcal sepsis | - |
| **Malignancies** | Multiple myeloma  Sigmoid colon carcinoma  Metastases of an unknown primary tumour | Non-melanoma skin cancer, twice in 1 patient | B-cell non-Hodgkin lymphoma | - |
| **Hospital admissions** | Resection sigmoid colon carcinoma, acute coronary syndrome, aortic root replacement, 2 myocardial infarctions, diarrhoea with dehydration, respiratory distress suspected to be due to pulmonary embolism and infection, haemolytic anaemia, pulmonary embolism, 2 total knee replacements, pyelonephritis, epileptic seizure, PCI for cardiac ischemia, surgery for spinal disc herniation, 3 admissions for constipation (in 1 patient), multiple sclerosis, motorbike accident. | PCI for cardiac ischemia, interstitial lung disease, total shoulder replacement. | Pulmonary embolism, total knee replacement, pneumonia, fever with high blood pressure and abdominal lymphadenopathy (unknown cause), stroke, surgery for a fractured ankle. | Polymyalgia rheumatica, septic arthritis of the left knee, bilateral extirpation of the adnexes (cyst). |

**Table S2. Serious adverse events during the second year of the IMPROVED-study**

OOP: outside of protocol, SAE: serious adverse event, PCI: percutaneous coronary intervention.
